# Reprogramming of Lipid Metabolism Mediates Crosstalk, Remodeling, and Intervention of Microenvironment Components in Breast Cancer

**DOI:** 10.7150/ijbs.92125

**Published:** 2024-03-03

**Authors:** Jia Wang, Wenfeng Zhang, Cun Liu, LongYun Wang, Jibiao Wu, Changgang Sun, Qibiao Wu

**Affiliations:** 1 State Key Laboratory of Quality Research in Chinese Medicines, Faculty of Chinese Medicine, Macau University of Science and Technology, Macau, China.; 2 College of First Clinical Medicine, Shandong University of Traditional Chinese Medicine, Jinan, China.; 3 College of Traditional Chinese Medicine, Shandong University of Traditional Chinese Medicine, Jinan, China.; 4 College of Traditional Chinese Medicine, Shandong Second Medical University, Weifang, China.; 5 Department of Oncology, Weifang Traditional Chinese Hospital, Weifang, China.

**Keywords:** Lipid metabolism, Breast cancer, Tumor microenvironment, Targeted intervention

## Abstract

Due to the unique characteristics of breast cancer initiation sites and significant alterations in tumor metabolism, breast cancer cells rely on lipid metabolic reprogramming to effectively regulate metabolic programs during the disease progression cascade. This adaptation enables them to meet the energy demands required for proliferation, invasion, metastasis, and responses to signaling molecules in the breast cancer microenvironment. In this review, we comprehensively examined the distinctive features of lipid metabolic reprogramming in breast cancer and elucidated the underlying mechanisms driving aberrant behavior of tumor cells. Additionally, we emphasize the potential role and adaptive changes in lipid metabolism within the breast cancer microenvironment, while summarizing recent preclinical studies. Overall, precise control over lipid metabolism rewiring and understanding of plasticity within the breast cancer microenvironment hold promising implications for developing targeted treatment strategies against this disease. Therefore, interventions targeting the lipid metabolism in breast cancer may facilitate innovative advancements in clinical applications.

## Background

With a deeper comprehension of the biological intricacies of breast cancer (BC), along with the intrinsic attributes of BC cells, there has been an increasing focus on the external factors that drive its onset and progression. A wealth of epidemiological evidence demonstrates a positive correlation between obesity (defined as BMI ≥ 30 kg/m^2^) and the risk of BC, distant metastasis, and specific mortality, particularly among postmenopausal women who are obese 1-4. The relationship between obesity and BC is complex because of the diverse sites and levels of estrogen biosynthesis before and after menopause. Although studies have found that premenopausal obese women seem to be associated with a reduced risk of BC, the inverse association between increased BMI and BC risk is most significant in early adulthood (ages 18-24), which may be due to premenopausal women whose estrogen is primarily derived from the ovaries, and the higher incidence of irregular menstrual cycles or anovulation in obese women, resulting in reduced exposure to estradiol and progesterone during the ovulation cycle 5, 6. The adipose tissue, which serves as the primary site for postmenopausal conversion of androgens into estrogen, elevates estrogen levels in obese women, thereby exposing them to an estrogen-rich tumorigenic environment 7. However, research has indicated that postmenopausal triple-negative breast cancer (TNBC) incidence and progression may be positively influenced by obesity 1.

Obesity has a discernible impact on the clinical management of BC, with significantly higher efficacy observed in radiotherapy, chemotherapy, and endocrine therapy among BC patients with normal BMI than in those who are obese 8, 9. Moreover, previous studies have demonstrated a significant correlation between obesity and larger BC tumors, positive lymph nodes, and shorter progression-free and overall survival 1, 10. Obesity is usually characterized by adipocyte hypertrophy, rapid adipose tissue remodeling, lipid metabolism disorders, etc., which can lead to hypoxia state, induce pro-inflammatory response to activate macrophages, and thus lead to local and systemic inflammation. In breast tissue from obese BC patients, dying fat cells surrounded by macrophages, called coronary structures, are observed under a microscope exhibiting increased aromatase activity 11. Meanwhile, high levels of TNF-α and IL-6 mediate insulin resistance and up-regulate insulin-like growth factor 1 (IGF-1) levels. IGF-1 is a potent mitogen of BC and is regulated by overexpressed IGF-1 receptor, which is significantly associated with poor prognosis of BC 12. The interaction between adipose cells, the predominant cell population in the breast, and ductal epithelial cells is a pivotal factor of BC progression.

Under normal physiological conditions, the interplay between adipocytes and epithelial cells within the mammary glands is constrained by the basement membrane barrier. However, upon malignant transformation, epithelial cells gain the ability to breach this barrier and become enveloped by an abundant pool of adipocytes that respond to their secretory milieu, comprising lipid metabolites, cytokines, and adipokines. This reciprocal communication fosters a conducive microenvironment for promoting BC development while concurrently inducing partial phenotypic and functional alterations in adipocytes 13, including 1)lipolysis-induced release of free fatty acids (FFAs) results in reduced volume; 2)enhanced secretion function promotes the mobilization of leptin, adiponectin, IL-6, TNF-α, and other adipokines and pro-inflammatory cytokines; 3)increased interaction between adipocytes and tumor cells regarding lipid metabolism has been observed. Studies have demonstrated the simultaneous enhancement of BC cell lipid metabolism and adipocyte lipolysis to meet the high energy demands of tumor tissues 14. This process implies that BC sustains its proliferative capacity through the lipid metabolic reprogramming (LMR), leading to alterations in metabolites with a cascade of biological effects conducive to its progression, such as the biosynthesis and oxidative decomposition of molecules such as FAs and cholesterol. Therefore, elucidating the mechanisms underlying lipid reprogramming in BC may offer an effective avenue for developing targeted intervention strategies for this disease.

Lipids serve not only as a pivotal energy source, but also as a fundamental substrate for biofilm formation and can function as signal transduction molecules to actively participate in diverse intracellular and extracellular information transmission processes 15. Tumor tissues undergo lipid remodeling by aberrantly augmenting multiple pathways, including de novo lipid synthesis, exogenous uptake, lipid droplet (LD) storage, and lipid oxidation, thereby ensuring the structural diversity of lipid pools and their long-term maintenance in an environmentally dependent manner 16. It is worth noting that to date, no studies have demonstrated differences in the types and functions of lipids derived from tumor-associated adipocyte secretion, blood circulation transport, and self-synthesis of tumor and non-tumor cells during tumor tissue metabolism. The underlying mechanism driving specific lipids within related pathways in BC is intricate and may be associated with molecular subtypes 17, 18. Furthermore, this process is closely associated with gene mutations, mitochondrial damage, and the BC microenvironment, particularly through its effects on the recruitment and functionality of stromal cells, immune cells, and other components within the BC microenvironment 19, 20.

In contrast to monoclonal cell populations, dynamic interactions between BC cells and non-tumor components drive high intratumoral heterogeneity, including microenvironmental heterogeneity, as they continuously evolve to adapt to different stages of tumor development 21. Therefore, targeting the cross-dialogue of lipid metabolism patterns and related metabolites among different cell components may hold potential for interfere with the tumor immune response. Given that a comprehensive description of the complete set of biochemical reactions involved in lipid metabolism has been extensively presented elsewhere 22, we aimed to systematically review the characteristics of LMR in BC and elucidate the mechanisms underlying the aberrant activity of tumor cells while discussing their variations across different subtypes. We observe the potential influence of lipid metabolism on the BC microenvironment, encompassing the pleiotropic effects on various components and adaptive changes arising from microenvironmental heterogeneity. Additionally, we provide the recent preclinical research on targeted interventions in lipid metabolism in BC. Our aim was to offer a molecular perspective on the interplay between lipid metabolism and the BC microenvironment, thereby facilitating the development of targeted therapeutic strategies for interventions targeting in lipid metabolism in this disease.

## Lipid metabolism landscape of breast cancer

**Figure 1:**
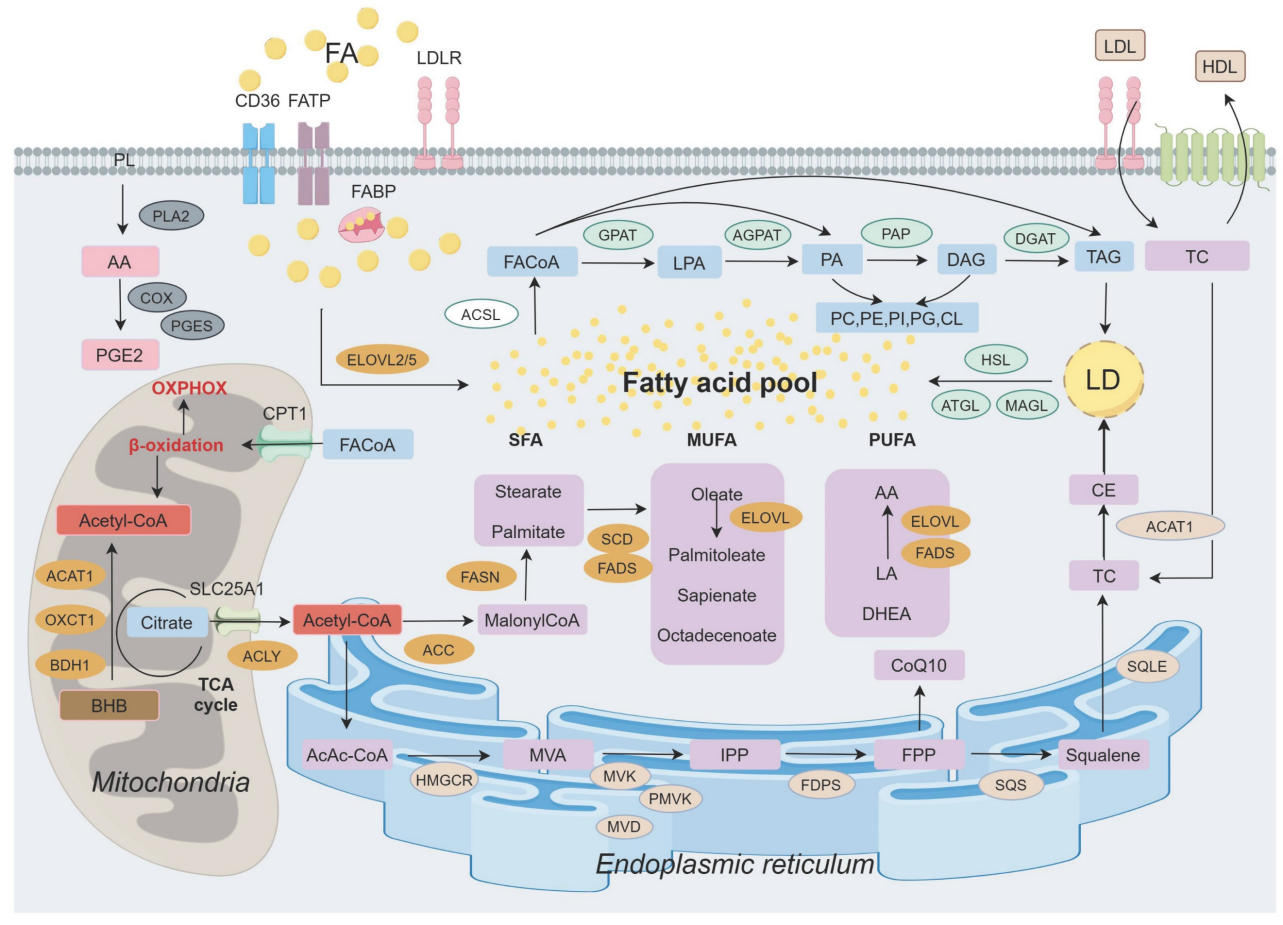
Landscape of lipid metabolism in breast cancer. The release of arachidonic acid (AA) by phospholipid (PL) on the cell membrane is implicated in prostaglandin (PG) synthesis. Exogenous fatty acids (FAs) enter the cellular lipid pool through various transmembrane transporters. Acetyl-CoA, produced in mitochondria from glucose, ketone bodies, and citrate, subsequently translocates to the cytoplasm for de novo FA synthesis before participating in triglyceride (TAG) synthesis following activation. Furthermore, FA within the endoplasmic reticulum contributes to cholesterol synthesis. Ultimately, FA is stored as TAG and cholesterol ester (CE) within lipid droplets (LDs) and can be mobilized to provide energy when required. Abbreviations: PLA2: Phospholipase A2; COX: Cyclo-oxyganese; BHB: β-hydroxybutyrate; PGES: Prostaglandin E2 synthase; PGE2: Prostaglandins E2; CPT1: Camitine palmitoyltransferase 1; ACAT1: Acetyl-CoA acetyltransferase 1; OXCT1: 3-succinyl coenzyme A transferase 1; BDH1: 3-hydroxybutyrate dehydrogenase 1; FACoA: Acyl-CoA; ACLY: ATP citrate lyase; ACC: Acetyl-CoA carboxylase; FASN: Fatty acid synthase; SFA: Saturated fatty acid; MUFA: Monounsaturated fatty acid; PUFA: Polyunsaturated fatty acid; SCD: Stearoyl coA desaturase; FADS: Fatty acid desaturase; ELOVL: Elongation of very long chain fatty acid; LA: Linoleic acid; DHEA: Docosahexaenoic acid; HMGCR: 3-hydroxy-3-methylglutaryl-CoA reductase; MVA: Mevalonate; MVK: Mevalonate kinase; PMVK: Mevalonate phosphate kinase; MVD: Mevalonate diphosphate decarboxylase; IPP: Isopentenyl pyrophosphate; FDPS: Farnesyl diphosphate synthase; FPP: 15-carbon pyrophosphate farnesylate; SQS: Squalene synthase; SQLE: Squalene epoxidase; TC: Total cholesterol; FATP: Fatty acid transport protein; FABP: Fatty acid binding protein; LDL: Low density lipoprotein; HDL: High density lipoprotein; ACSL: Acyl-coenzymeA synthetase; GPAT: Glycerol-3-phosphate acyltransferase; LPA: Lysophosphatidic acid; AGPAT: Acylglycerol-3-phosphate acyltransferase; PA: Phosphatidic acid; PAP: Phosphatidic acid phosphatase; DAG: Diacylglycerol; DGAT: Diacylglycerol acyltransferase; PC: Phosphatidylcholine; PE: Phosphatidyl ethanolamine; PI: Phosphatidylinositol; CL: Cardiolipin; HSL: Hormone-sensitive lipase; ATGL: Adipose triglyceride lipase; MAGL: Monacylglycerol lipase.

Lipid, metabolism undergoes dynamic changes in response to tumor status, characterized by alterations in lipid synthesis, uptake, storage, catabolism, various metabolites, and catalytic enzymes (Figure 1). In addition to its established functions, it exerts aberrant effects that support malignant transformation, including the upregulation of FA levels, enhanced oxidative decomposition, and increased demand for biofilm formation. It significantly promotes the progression of different BC subtypes.

The elevation of FA levels in BC is contingent on augmented exogenous uptake and storage capacity. Notably, various tumors, including BC, exhibit upregulation of specialized transporters such as membrane glycoprotein CD36 and fatty acid binding proteins (FABPs) 2. FABP4 not only facilitates FA uptake and transportation but also enhances the expression levels of CD36 and FABP5, thereby promoting BC proliferation 17. Balaban et al. demonstrated that in a lipid-rich extracellular environment, high extracellular lipid availability further enhances FA flux in BC cells, and elevated palmitate levels induce lipid toxicity, at which point exogenous FA is accumulated to be converted into triglycerides and stored in LDs to protect BC cells from palmitate-induced apoptosis. Compared with MCF-7 cells, MDA-MB-231 cells had lower fatty acid oxidation (FAO) and triglyceride hydrolysis rates and stronger triglyceride synthesis ability 23. In contrast, the de novo synthesis pathway played a more significant role. ATP citrate lyase (ACLY), acetyl-CoA carboxylase (ACC), and fatty acid synthetase (FASN) exhibit significantly higher expression levels in HER2+ BC, whereas their prominence is not observed in TNBC where their expression level is extremely low 24. In TNBC, the inhibition of FASN expression can overcome chemotherapy resistance in tumor cells, demonstrating a consistent indirect anti-tumor effect 25. Upregulation of FASN in other subtypes can lead to metastatic brain damage in BC; however, targeted disruption of this program can prevent BC brain metastasis while maintaining lipid synthesis and palmitic acid (a saturated fatty acid, SFA) levels, even in the absence of exogenous lipids 26, 27. ACC is upregulated in early stage BC and its inhibition significantly reduces FA synthesis. ACC phosphorylation is associated with BC metastasis 28, 29. Inhibition of ACC and FASN expression induces apoptosis in BC cells. This may be attributed to AMP-activated protein kinase (AMPK) inhibits the activity of ACC1 and ACC2 by phosphorylating them, thereby suppressing malonyl-CoA production and leading to lipid peroxidation (LPO) 29-31.

Moreover, the regulation of key enzymes involved in the de novo synthesis of FAs is mediated by a diverse array of signaling molecules. Sterol regulatory element binding protein 1 (SREBP1) serves as a pivotal transcription factor that stimulates the expression of crucial enzymes including ACLY, ACC1, FASN, and stearoyl-CoA desaturase 1 (SCD1). Its overexpression has been linked to an unfavorable prognosis in BC and can be activated by both the mTOR complex 1 (mTORC1) and mTOR complex 2 (mTORC2) 32-35. Specifically, mTORC2 phosphorylates and may activate ACLY, thereby promoting the proliferation of HER2+ and PI3K mutated BC cells 36, 37. Additionally, MYC has been implicated in the upregulation of circulating tricarboxylic acid enzymes and enzymes related to FA synthesis. Previous studies have demonstrated that aspirin and compound C (an AMPK inhibitor) inhibit the expression of FA synthesis-related enzymes via C-MYC, thereby impeding HER2+ BC 38, 39. Upon ubiquitin modification, the tumor suppressor gene PTEN interacts with FASN within the nucleus, leading to opposing effects by attenuating FASN phosphorylation and ubiquitin modification. This interaction weakens the association between FASN and the ubiquitin ligase TRIM21 while enhancing the protein stability of FASN. Consequently, the de novo synthesis pathway for FAs is augmented, promoting the malignant progression of BC 40.

FAO and lipolysis are pivotal pathways that provide energy to BC cells, and dysregulation of these processes can induce lipid accumulation leading to cell death. FAO is frequently observed as a prominent metabolic feature of TNBC 41. Acyl-coA synthase 4 (ACSL4) and carnitine lipoacyltransferase 1A (CPT1A), which are associated with poor prognosis, are overexpressed in invasive and recurrent BC, respectively 42, 43. Acyl-coa oxidase 2-i9 (ACOX2-i9), a variant transcript of the rate-limiting enzyme ACOX2 involved in FA β-oxidation, exhibits specific enrichment in ER+ BC; its knock-down results in reduced tumor cell viability 44. The key enzymes involved in lipolysis, namely adipose triglyceride lipase (ATGL) and monacylglycerol lipase (MAGL), have been found to be significantly upregulated in BC, and their overactivation is associated with invasion 45-47. Specifically targeting the rate-limiting enzyme ATGL for neutrophil triglyceride degradation enhances lipid accumulation in the lungs and promotes lung metastasis in BC 48. Studies have demonstrated that peroxisome proliferator activated receptor-α (PPAR-α) agonists promote lipid oxidation by upregulating CPT1A while downregulating the expression activities of ACOX and glutathione peroxidase 4 (GPX4). Additionally, they induce ATGL-mediated lipolysis through processes such as autophagy and oxidative stress 49-51. Furthermore, PPAR-γ is implicated in the ATGL-mediated lipolysis pathway, and BC cytokines downregulate the transcriptional activity of PPAR-γ to induce mitochondrial dysfunction and lipid accumulation in skeletal muscle 52, 53.

Abnormal proliferation of BC cells necessitates the involvement of biofilms in the synthesis and metabolism of phospholipids, cholesterol, and sphingolipids. Notably, Lipin-1 (a phosphatidyl phosphatase) and GPAT2 exhibited significant expression in BC. The proto-oncogene Src mediates the phosphorylation of Lipin-1 to upregulate phospholipid and triglyceride synthesis to sustain proliferation; knockdown of Lipin-1 inhibits both the proliferation and migration of BC 54-56. Additionally, Lipin-1 acts as a negative regulator of SREBP1, whereas mTORC1 promotes SREBP1 expression by impeding the nuclear entry of Lipin-1 57. In terms of lipid co-expression within the context of BC, remarkable overexpression was observed in ether lipids within the SKBr3 cell line (estrogen receptor-negative but G-protein-coupled estrogen receptor-1 positive), accompanied by elevated sphingolipid levels 58, indicating potential risks associated with lipid imbalance. Furthermore, an increased copy number at the squalene monooxygenase (SQLE) loci contributes to excessive cholesterol production in BC, which significantly correlates with tumor progression and poor prognosis 59. Phosphorylation of hydroxymethylglutarate monoacyl-CoA reductase (HMGCR) and hormone-sensitive lipase (HSL) by AMPK, as well as their subsequent inactivation, play a pivotal role in the inhibition of cholesterol synthesis and lipolysis in BC 60, 61. The conversion of inactive cholesterol into cholesterol esters is facilitated by acetyl-CoA acetyltransferase (ACAT1), with the accumulation of cholesterol esters within LDs promoting BC proliferation and invasion. Moreover, elevated ACAT1 expression is positively associated with the low-density lipoprotein-mediated proliferation observed in hormone receptor-negative BC, showing increased cholesterol esters 62-64.

Additionally, lipids, including cholesterol, play a crucial role in the biosynthesis of steroid hormones such as estrogen, progesterone, and testosterone. Consequently, cholesterol metabolism may exhibit significant variations among different molecular subtypes of BC. The liver X receptor (LXR), a pivotal transcription factor involved in this process, effectively inhibits proliferation in ER+ and basal-like BC cells 65. Activation of LXR by its ligand T0901317 suppresses the expression of Flotillin-2, a biomarker associated with lipid rafts 66. Another ligand for LXR is 27-hydroxycholesterol (27-HC), which acts as a selective estrogen receptor agonist and promotes the proliferation of MCF-7 cells (ER+). Notably, high expression levels of its metabolic enzyme CYP7B1 are indicative of improved disease-free survival rates in patients with ER+ BC. Furthermore, downstream metabolites were significantly enriched in hormone receptor-positive BC 67. TNBC exhibits elevated levels of phosphatidylcholine (PC) compared to ER+ BC, which is potentially associated with the generation of high- and low-density lipoproteins during cholesterol metabolism 68. The aforementioned aberrant alterations in lipid metabolism collectively mobilize lipid synthesis and utilization, thereby triggering a series of aberrant activities of BC cells.

## Dysregulated lipid metabolism drives aberrant behavior in breast cancer cells

**Figure 2:**
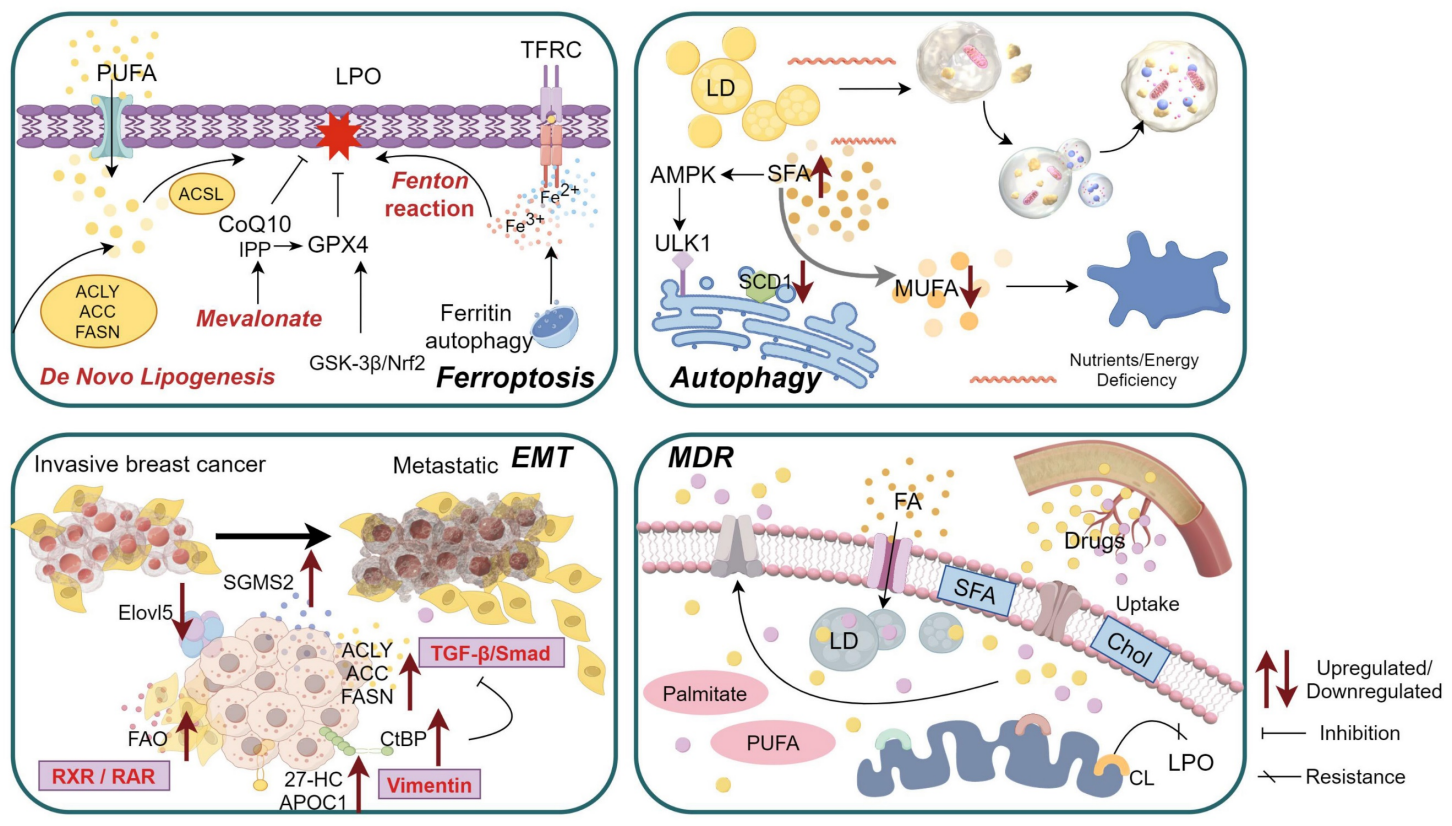
Aberrant activity of breast cancer (BC) cells driven by lipid metabolism reprogramming. 1)Exogenous fatty acid (FA) uptake, de novo FA synthesis, mevalonate, and fenton reaction collectively contribute to lipid peroxidation (LPO), ultimately inducing ferroptosis; 2)The dual effect of SCD1-mediated autophagy is closely associated with specific types of FAs and lipid toxicity, while the autophagic initiation of lipid droplets (LDs) triggered by the low-pH and nutrients/energy deficiency microenvironment; 3)During invasive BC metastasis through epithelial-mesenchymal transition (EMT), several factors related to lipid metabolism undergo alterations in both tumor cells and stromal cells; 4)Tumor cells promote multidrug resistance (MDR) by reducing drugs uptake and increasing drugs efflux, the increase of cardiolipin (CL) on the mitochondrial membrane in drug-resistant cells leads to the increase of LPO resistance, hydrophobic cytotoxic drugs can be sequestered within enlarged LDs, thereby impeding their efficacy. Abbreviations: PUFA: Polyunsaturated fatty acid; TFRC: Transferrin receptor; SFA: Saturated fatty acid; IPP: Isopentenyl pyrophosphate; MUFA: Monounsaturated fatty acid; FAO: Fatty acid oxidation; Chol: Cholesterol.

The functional and biological behavior of BC cells is influenced by the state transition, which is characterized by the LMR. Lipid metabolism undergoes broad enhancement throughout the different stages of BC, serving as both an energy source for tumor cells and a trigger for specific signaling pathways and aberrant cellular activity (Figure 2).

### Ferroptosis

LPO induced by high levels of reactive oxygen species (ROS) has recently been shown to mediate an iron-dependent form of programmed cell death known as ferroptosis. Unlike other modes of cell death that are prone to resistance, thier distinctive features include iron accumulation and LPO, making targeted intervention and induction promising avenues for designing BC therapy. Iron accumulation subsequently triggers LPO through a non-enzymatic iron-dependent Fenton reaction and the activation of iron-containing enzymes, such as lipoxygenase 69, 70.

In BC, the cell death mode of the antioxidant system leads to reduced expression of GPX4 71, a central regulator that oxidizes glutathione, reduces cytotoxic lipid peroxides, and inhibits lipid peroxide production and ferroptosis. The mevalonate (MVA) pathway biomolecule isopentenyl pyrophosphate (IPP) and coenzyme Q10 play a role in ferroptosis resistance, with IPP being necessary for GPX4 biosynthesis 72. GPX4 can induce the expression of the pentaspanin protein prominin-2, which facilitates the formation of exosomes containing ferritin. This mechanism promotes resistance to ferroptosis in both breast epithelial and BC cells 73. Notably, GPX4 expression has demonstrated significant predictive value in neoadjuvant therapy for BC, and high GPX4 expression is positively associated with distant metastasis-free survival 74. Studies have revealed that glycogen synthase kinase-3β (GSK-3β) can disrupt cellular antioxidant defense by modulating nuclear factor erythroid 2-associated factor 2 (Nrf2). Reduced GSK-3β expression in BC leads to upregulation of GPX4 and downregulation of arachidonic acid 15-lipoxygenase, resulting in decreased levels of ROS and malondialdehyde (MDA). Ferroptosis induced by GSK-3β overexpression can be attenuated by Nrf2, suggesting that targeting the GSK-3β/Nrf2 axis may hold promise for inhibiting tumor growth 75. In addition, heme oxygenase (HO) serves as the primary target protein of Nrf2, plays a crucial role in heme catabolism, and is closely associated with iron release. Notably, HO-1 exerts a bidirectional effect on tumors; thus, the inhibition of HO-1 is widely recognized as an effective strategy for tumor therapy. The emerging correlation between HO-1 expression and ferroptosis suggests that cells with lower HO levels are more susceptible to ferroptosis. Iron supplementation and enzyme induction can accelerate Erastin-induced ferroptosis 76.

Yi et al. and Luis et al. demonstrated that SCD1 plays a key role as a regulator of ferroptosis. Activation of the PI3K-AKT-mTOR pathway promotes cell migration through downstream SREBP1 up-regulation of SCD1 expression, mediates increased monounsaturated fatty acid (MUFA) synthesis (increased MUFA/ polyunsaturated fatty acids (PUFA) ratio), and protects BC cells from ROS-induced ferroptosis 77, 78. In contrast to PUFA, MUFA inhibits LPO and ferroptosis by displacing PUFA from phospholipids in cell membranes. Therefore, inhibition of SCD1 can induce ferroptosis by sensitizing tumor cells to ROS and ferroptosis inducers. Interferon-secreted by CD8+T cells and arachidonic acid induction within the tumor microenvironment (TME) facilitate ferroptosis through ACSL4-dependent LMR 79. Fascin actin-binding protein 1 was significantly upregulated in tamoxifen-resistant BC cells, thereby enhancing their susceptibility to erastin-induced ferroptosis 80. Therefore, targeting the induction of ferroptosis in drug-resistant cells may represent a novel strategy to overcome drug resistance.

### Autophagy

Emerging evidence suggests that LMR optimize lipid configuration, rendering it a biosensor implicated in tumor autophagy. Studies have indicated that in a variety of tissues or cells, the up-regulation of FA levels will increase autophagy. For instance, high concentrations (at least 500μM) of unsaturated fatty acids (UFAs, such as oleic acid) show a significant promotion of autophagy. PUFA glycerol monoester docosahexaenoate induced apoptosis and autophagy in BC cells. Here, autophagy is an inhibitor of apoptosis 81. However, in the presence of high concentrations of SFAs (such as palmitic acid), autophagy is inhibited 82, probably because they are not efficiently converted into triglycerides stored in LDs. The desaturase SCD1 has a dual effect on the regulation of tumor cell autophagy, which may be related to FA type, lipid toxicity, and cell type. SCD1 converts SFAs into UFAs to protect tumor cells from lipotoxic damage. From the perspective of autophagy as a protective mechanism, reduced levels of SCD1 result in moderate accumulation of SFAs, triggering an AMPK-mediated compensatory resistance mechanism that accelerates the process of autophagy, prevents further FA accumulation, and thus escapes the cytotoxic effect caused by the increase of SFAs, and maintains cell survival 83. However, high levels of lipid toxicity, excessive autophagy, and consumption of MUFAs by inhibiting SCD1 can lead to cell death 84. Compared with differentiated cells, breast cancer stem cells (BCSCs) have a higher autophagy flux 85, inhibition of SCD1 makes them susceptible to overactivation of autophagy to induce cell death. In summary, SCD1 exerts a strongly directional effect on autophagy through a specific balance between saturated and unsaturated fatty acids.

Autophagy is also associated with low-pH environments, membrane domains, and multiple lipid mediators in the blood. The activation of the highly expressed acid-sensitive receptor ovarian cancer G protein-coupled receptor 1 (OGR1) in BC triggers the accumulation of LDs derived from ketogenic amino acids during autophagy. Given its involvement in endoplasmic reticulum stress-induced autophagy under acidic conditions, depletion of OGR1 not only reduces acid-induced lipid accumulation but also inhibits cell proliferation within an acidic microenvironment while impairing the endoplasmic reticulum stress response and initiation of autophagy 86. Abnormal expression of CAV1, a marker for lipid raft/cholesterol-rich membrane microdomains (CEMM), has also been observed in BC, with reduced expression levels. CEMM functions as a barrier that restricts the activity of the v-SNARE protein vesicle-associated membrane protein 3 (VAMP3) during autophagy-vesicle fusion. Additionally, it facilitates the interaction between VAMP3 and STX6 (a cholesterol binding SNARE protein). The absence of CEMM leads to the dissociation of VAMP3 from STX6, triggering autophagy in BC cells and contributing to doxorubicin (DOX) resistance 87. Sphingodylcholine (SPC), a lipid mediator present in the blood, mediates both autophagy and apoptosis in TNBC cell lines; however, autophagy acts as a negative regulator of SPC-induced apoptosis. SPC induces apoptosis by activating the autophagy/AKT/p38 signal transduction pathway and antagonizing c-JNK signal transduction 88.

Under normal circumstances, lipolysis refers to the intracellular release of FAs from LDs through the activation of enzymes, such as ATGL, HSL, and MAGL. Recent evidence suggests that lipid autophagy serves as an alternative pathway for lipolysis. In MDA-MB-231 cells, PUFAs such as docosahexaenoic acid (DHEA) have been found to induce lipid autophagy, which is further enhanced by the combined action of vitamin E and delta-tocotrienol resulting in reduced formation of LDs 89. Moreover, DHEA has been shown to promote BC autophagosome formation through two mechanisms: 1) up-regulation of oxidative stress-induced growth inhibitor 1 expression by DHEA temporarily increases mitochondrial ROS levels and subsequently activates p-AMPKα to facilitate autophagosome formation 90; 2) PFAs such as DHEA and eicosapentaenoic acid induce phosphorylation of Bcl-2 leading to dissociation from beclin-1 and subsequent induction of BC cell autophagy 91. The regulatory role of DHEA in BC autophagy merits further exploration and consideration.

### Epithelial-mesenchymal Transition (EMT)

It is widely acknowledged that most BC cells originate from epithelial cells. Epithelial cells play a crucial role in mediating various phenotypic changes during their transformation into an interstitial phenotype under specific conditions, including alterations in cell morphology, weakened or lost adhesion, and enhanced stem cell-like characteristics. These changes are extensively involved in the initiation, invasion, and metastasis of BC, as well as in the development of chemotherapy resistance. Reversal of the phenotype through mesenchymal-epithelial transition (MET) is possible. The intricate diversity of lipid metabolic pathways and intermediates contributes to the complex mechanisms underlying EMT in BC. SREBP1 downregulates E-cadherin expression by recruiting the Snail/HDAC1/2 complex and miR-18a-5p has been identified as a potential target of SREBP1 regulation. Overexpression of SREBP1 and inhibition of miR-18a-5p significantly induces BC metastasis 92. Additionally, sphingomyelin synthase 2 overexpressed in BC acts as a key regulator influencing sphingomyelin (SM) homeostasis and activates TGF-β/Smad signaling pathway to initiate EMT by enhancing TGF-β1 activity level thereby promoting invasion and metastasis of BC cells 93.

The disparity in FA metabolic requirements between epithelial cells and mesenchymal cells may serve as a pivotal factor in achieving the MET phenotype. Notably, the expression of elongation of very long-chain fatty acids protein 5 (Elovl5) was significantly downregulated in metastatic estrogen receptor-positive BC, exhibiting a positive correlation with EMT, invasion, and lung metastasis. Mechanistically, reduced Elovl5 expression promotes upregulation of TGF-β receptor signaling mediated by Smad2 acetylation dependent on LD accumulation 94. Extracellular vesicles derived from TNBC cells stimulated with PFAs enhance the expression of multiple EMT-related genes including Snail1, vimentin, MMP-2 and -9 secretions 95. In addition, mesenchymal cells are associated with increased FAO levels. FAO facilitates histone acetylation modifications in EMT genes through an acetyl-CoA-dependent pathway, thereby influencing the epigenetic regulation of EMT. Alterations in FAO redirect FAs towards lipid storage in epithelial cells via RXR/RAR signal transduction to promote a more pronounced epithelial cell phenotype, whereas inhibition of key enzymes disrupts MET 96.

As a constituent of the cytoplasm and cell membrane, cholesterol plays a direct or indirect role in regulating the molecular markers of EMT. Additionally, it influences EMT by modulating cell membrane fluidity. ZMYND8 functions as an epigenetic enhancer and is partially expressed in BCSCs. ZMYND8 enhances intracellular accumulation and oxidation of cholesterol while inhibiting the catabolism of 27-HC. Elevated levels of 27-HC activate LXR and suppress the expression of E-cadherin and β-Catenin. This leads to upregulation of Snail1, Vimentin, fibroblast activating protein α, MMP9, and STAT3 at both transcriptome levels and activities 97-99. Apolipoprotein C1 employs a similar mechanism and participates in the EMT of BC by suppressing E-cadherin and promoting vimentin expression 100. The upregulation of niacinamide N-methyltransferase in TNBC downregulates protein phosphatase 1A activity, thereby activating the MEK/ERK/c-Jun/ABCA2 signaling pathway to facilitate cholesterol efflux and enhance cell membrane fluidity, ultimately inducing EMT and TNBC metastasis 101. The co-repressor CtBP, which is highly expressed in BC, modulates intracellular cholesterol homeostasis by inhibiting SREBF2 and HMGCR activity, Decreased cholesterol levels can compromise TGF-β receptor stability on the cell membrane, consequently triggering EMT and facilitating cancer cell metastasis 102.

### Multidrug Resistance (MDR)

In addition to indirectly contributing to chemotherapy resistance by promoting EMT in BC, the development of the MDR phenotype is a direct consequence of alterations in lipid metabolite levels, creating favorable conditions for the survival of BC cells. Changes in the lipid composition of the cell membrane, activation of FAO, synthesis of FAs, enhanced uptake, and abnormal accumulation of lipids may confer resistance to stressors in BC cells 103, involving intricate mechanisms: 1) The cell membrane exhibits an abundance of long-chain SFAs with a dense arrangement. Elevated cholesterol levels decrease membrane fluidity and potentially limits drug uptake. For instance, the concentrations of sphingomyelin, phosphatidylinositol, cholesterol and cholesterol esters in the lipids of drug-resistant BC cell membranes were higher than those of sensitive cell membranes, decreased cholesterol content in the cell membrane of MCF-7 BC cells enhances DOX uptake 104. 2) Changes in the distribution of lipids in the plasma membrane of drug-resistant cells, as well as the mutual transformation between the plasma membrane and the organelle membrane, lead to changes in the lipid profile of the organelles in drug-resistant cells to maintain MDR, including the lipid profile of the mitochondrial membrane 105. Cardiolipin (CL), which mainly exists on the mitochondrial membrane, is easily oxidized by reactive oxygen species, resulting in cytochrome A-dependent cell apoptosis. Less oxidizable CL can reduce cell apoptosis. Some studies have found that the increase of CL in DOX or cisplatin resistant MCF-7 cells leads to increased resistance to LPO, possibly because of the existence of CL species with higher resistance to LPO (e.g. with a higher saturated/unsaturated fatty acyl ratio) 106. 3) Increased exogenous intake of FAs resulted in elevated LDs and triglyceride levels. Hydrophobic cytotoxic drugs such as docetaxel and tamoxifen can be easily sequestered within LDs 107, 108. Furthermore, the upregulation of CD36, an FA transporter, has been observed in HER2 inhibitor lapatinib-resistant cells 109. 4) Specific precursors of certain lipid components can act as second messengers to activate multiple signaling cascades and induce the transcriptional expression of drug efflux transporter genes. Multiple ligands for free fatty acid receptor 4 (FFAR4), including stearic acid, docosatetraenoic acid, docosapentaenoic acid and DHEA are increased in BC. These ligands induce tamoxifen resistance in hormone receptor-positive BC by activating the ERK and AKT pathways 110.

A range of crucial genes implicated in drug resistance can interact with specific lipids, particularly those highlighted by significant secondary messenger molecules within sphingomyelin metabolic pathways. Reprogramming sphingosphingoid metabolism in DOX-resistant cells tends to decrease ceramide (Cer) levels and increase SM levels, enabling evasion of ceramide-induced apoptosis through activation of the SM synthesis pathway. Increased SM content can recruit and functionalize ABCB1, promoting a partial multidrug resistant state in cells, along with the accumulation of glucosylceramide and sphingolipid 111, 112. The Cer derivative sphingosine has been identified as a potential molecular target for characterizing chemotherapy resistance in sphingoid metabolism, in BC as its activity level is regulated by sphingosine kinase 1 113, 114. Additionally, MCF-7 and MDA-MB-231 cell lines, which are resistant to paclitaxel (TAX), exhibit similar patterns of PC biosynthesis and are associated with poor prognosis compared with the sensitive cell lines MCF-7, BT474, and HER2+ 115. Different subtypes of BC display variations in lipid metabolism among drug-resistant cells, characterized by distinct gene expression patterns. Activation of JAK/STAT3-regulated FAO can enhance paclitaxel resistance in mouse mammary stem cells 116. The activity levels of FASN and CPT1A are positively correlated with chemotherapy resistance in HER2+ and hormone receptor-positive BC, respectively 117-119.

## The interplay between lipid metabolism reprogramming and the tumor microenvironment in breast cancer

**Figure 3:**
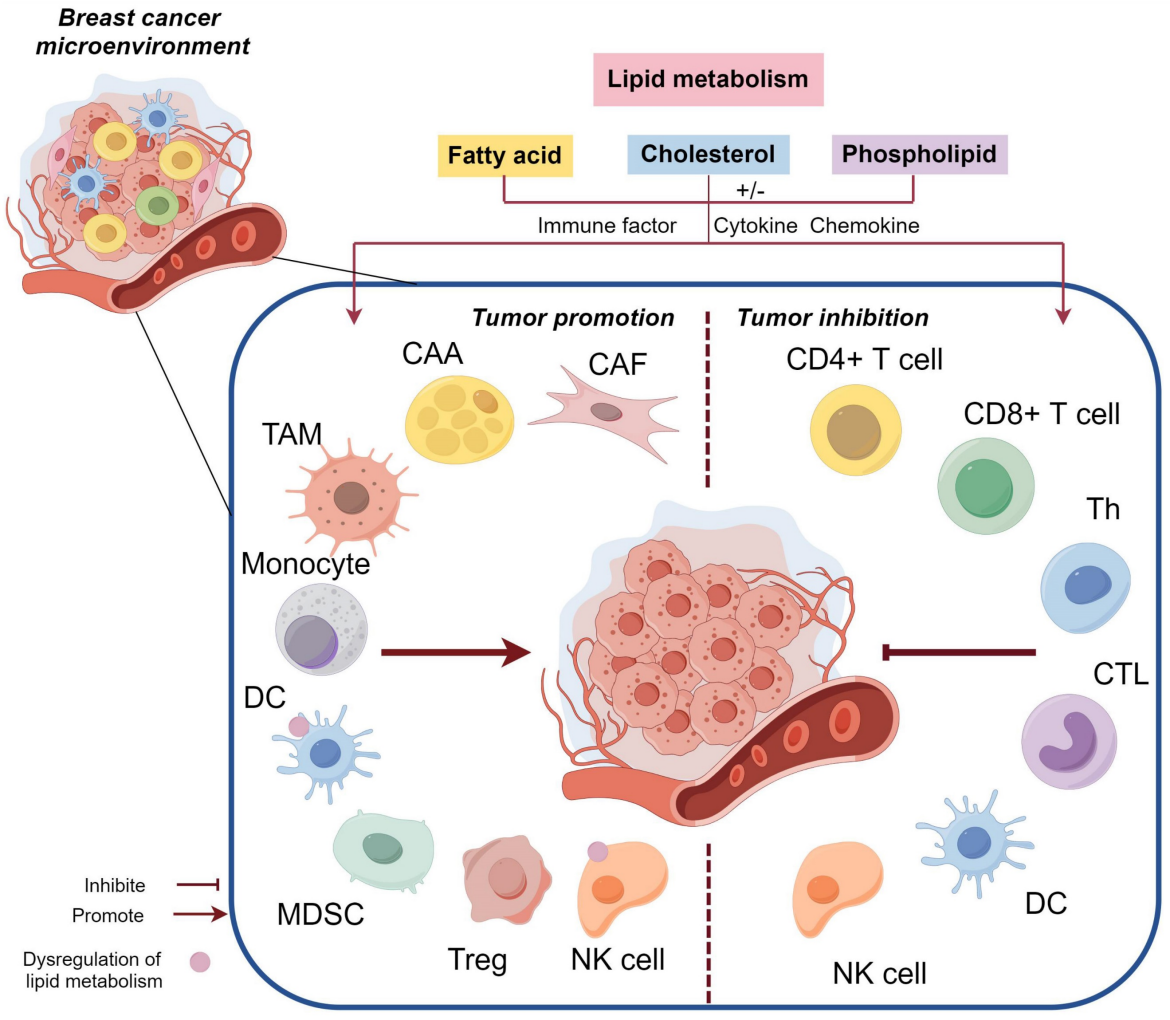
The impact of lipid metabolism on the tumor microenvironment in breast cancer (BC). Lipid metabolism facilitates the involvement of CAFs, CAAs, TAMs, MDSCs, and Tregs in promoting BC through diverse mechanisms, while impairing the phenotype and function of DCs and NK cells, resulting in immunosuppression and even tumor promotion. Furthermore, lipid metabolism influences CD4+ T cells, CD8+ T cells, and other cell types to exert an anti-BC effect.

**Figure 4:**
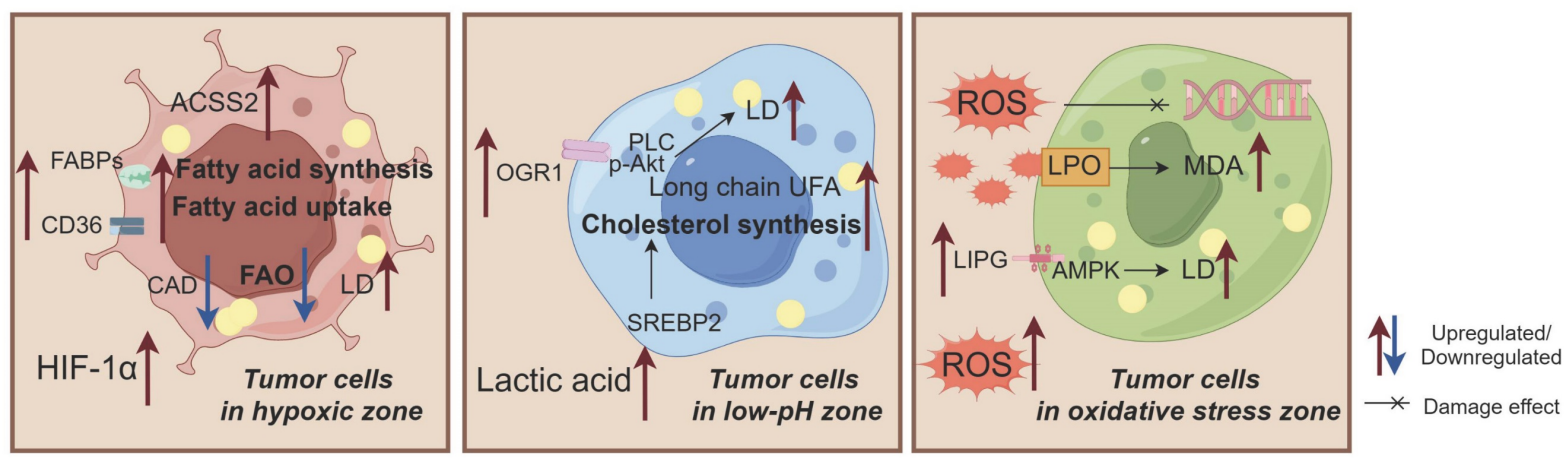
The adaptive alterations in lipid metabolism observed in breast cancer (BC) cells exposed to hypoxia, low pH, and oxidative stress. 1)These conditions induce exogenous fatty acid (FA) uptake, enhance de novo synthesis, and weaken fatty acid oxidation (FAO), ultimately leading to lipid droplet (LD) accumulation and promoting BC growth; 2)Low pH conditions alter the tendency of long chain unsaturated fatty acids (UFAs), increase cholesterol biosynthesis, and promote LD accumulation; 3)Oxidative stress is characterized by free radical-induced oxidative damage that activates lipid peroxidation (LPO) and AMPK pathways while increasing malondialdehyde (MDA) levels and LD accumulation. Acyl-CoA dehydrogenase (CAD) plays a crucial role in these processes. Abbreviations: ACSS2: Acetyl-CoA synthase 2; FABP: Fatty acid binding protein; OGR1: Ovarian cancer G protein-coupled receptor 1; LIPG: Endothelial lipase.

The abnormal activity and biological behavior of BC cells driven by LMR are not solely attributed to a single factor, but rather arise from the interplay of multiple factors, including abundant secretory factors and crosstalk between multicellular components within the BC microenvironment. The dynamic regulation of lipid metabolism encompasses active cascades involving different cell populations and passive effects resulting from microenvironmental heterogeneity. LMR exert pleiotropic effects by modifying microenvironment constituents to promote BC progression (Figure 3). Moreover, the high heterogeneity of the BC microenvironment induces adaptive changes in lipid metabolism, potentially influencing its potiential role as a signaling entitiy in BC through the synthesis of lipid substrates (Figure 4).

### The pleiotropic role of lipid metabolism reprogramming in intercellular communication

#### Fibroblasts

Cancer-associated fibroblasts (CAFs), the most abundant cell type in the TME, are activated fibroblasts and are the center of cross-communication between various cellular components. CAFs consist of highly heterogeneous subpopulations with different or even opposite functions, and can be derived from inactive fibroblasts, adipocytes, endothelial cells, and stellate cells 120. Yu et al. proposed that ineffective clinical CAF-directed interventions can be circumvented by selectively targeting specific CAF subgroups. CD10, a transmembrane hydrolase expressed on the surface of CAF subgroups, can degrade the osteoblastic growth peptide (OGP), an anti-tumor peptide, to maintain the tumor stems of BCSC. As a key substrate of CD10, OGP reduces the production of monounsaturated FAs by inhibiting SCD1 expression, which regulates lipid metabolism in BC 121. When exposed to CAF, the activity of the lipid synthetase FASN in BC cells decreased, and the ability of lipid uptake increased, making FA transport protein 1 a key FA transporter, the expression of FABP2 and FABP3 decreased 122. The metabolic characteristics change from lipid producers to lipid collectors. Furthermore, it has been observed that G protein-coupled estrogen receptors upregulate the expression and activity of FASN in both BC cells and BC-associated fibroblasts, significantly promoting BC progression 123. Additionally, the overexpression and secretion of the aspartate protease cathepsin-D in BC cells stimulates fibroblast growth by binding to low-density lipoprotein receptor-associated proteins within the microenvironment 124. Moreover, dysregulated saturated lipids processing was found to be profibrotic 125 and induce pro-fibrotic fibroblast activation, which may contribute to radiation-induced fibrosis during radiotherapy for BC. Diacylglycerol kinase α plays a crucial role in maintaining diacylglycerol-mediated lipid homeostasis, and inhibiting its expression can effectively impede the process of fibrosis induced by radiotherapy 126.

#### Adipocytes

The paracrine interaction between epithelial cells and adipocytes in the breast tissue contributes to the increased malignant potential of BC through complex mechanisms, including metabolic remodeling, invasion, susceptibility to BC, and inflammation 127. Abnormal adipocytes that undergo phenotypic changes under the influence of tumor cells are referred to as cancer-associated adipocytes (CAAs), which exhibit microscopic characteristics such as smaller cell volume and LDs, fibroblast-like changes, and reduced lipid differentiation 128 due to an increase in mitochondrial FAO. Secreted FFAs induce LMR in BC cells by altering phospholipid organization in their membranes, particularly reducing arachidonic acid content (a type of PFA) leading to increased autophagy flux, maturation, and lysosomal acidification within these cells before inducing autophagy 129. In contrast, exposure to CAAs upregulates ATGL expression in BC cells while promoting lipolysis pathways that respond positively to enhanced FAO from tumors, which is correlated with tumor invasion and metastasis. Evidence suggests that adrenal medullin receptor activation induces UCP1 overexpression in the CAAs found within both the primary microenvironment of breast and metastatic niches in obese patients, further promoting HSL phosphorylation 45, 130. Fibroblast-like changes in CAAs are accompanied by the downregulation of terminal differentiation markers and the upregulation of pro-inflammatory cytokines (IL-6 and PLOD2), ultimately leading to the emergence of adipocyte-derived fibroblasts that may incorporate tumor stromal components 131-133. However, it should be noted that fibroblast-like cells within CAAs do not necessarily originate from adipocytes but can also represent stromal cells recruited by tumor cells. The absence of smooth muscle actin expression in adipose-derived fibroblasts may contribute to marker heterogeneity among BC-associated fibroblasts 133.

Various metabolic mediators released by adipocytes exert distinct effects on different BC cell subtypes. Adipose-derived oleic acid suppresses LPO in TNBC cells, thereby protecting them from ferroptosis through ACSL3 134. Interestingly, oleic acid sustains the malignant characteristics of highly metastatic BC cells by activating AMPK-mediated FA β-oxidation, while conversely inhibiting the proliferation of low-metastatic MCF-7 cell line 135. Adipocyte-released estrogen stimulates the division and proliferation of estrogen-sensitive BC cell subtypes 136. The production of inflammatory cytokines in breast adipocytes increases with postmenopausal and adipose tissue expansion, the interaction of inflammatory adipocytes with BC cells stimulates the production of estrone (postmenopausal estrogen) and NF-κB-dependent cytokine 137. Aromatase, as a rate-limiting enzyme of estrogen synthesis, is expressed in preadipocytes. Since breast preadipocytes are closer to mammary epithelial cells, their expression of aromatase is more effective in carcinogenic development than that expressed elsewhere, resulting in a significant increase in local estrogen 138. In addition, the expression of aromatase is regulated by many factors, such as cytokines and nuclear receptors. Type I interferons induce aromatase expression in preadipocytes around BC, and similarly, leptin signaling secreted by adipose cells increases aromatase expression, thus promoting ER+ BC progression 139, 140.

#### Immune cells

As the principal executors of immune function, immune cells can identify and eliminate aberrant cells during immune surveillance, thereby maintaining homeostasis. However, a diverse array of lipid metabolic intermediates and products can modulate the functional role of immune cells (both innate and adaptive) in BC's immune editing process in BC, subsequently exerting tumor immunosuppressive effects.

##### Innate immune cells (macrophages, natural killer (NK) cells, dendritic cells (DCs), marrow derived suppressor cells (MDSCs))

A diverse array of innate immune cells contributes to the establishment of an immunosuppressive microenvironment in BC, which is characterized by lipid dysregulation. Wu et al. demonstrated that enhancing the lipid metabolism pathway was sufficient to induce the conversion of macrophages into immunosuppressed tumor-associated macrophages (TAMs) 141. Activation of Caspase 1 in TAMs in a mouse model of BC upregulates medium-chain acyl-CoA dehydrogenase, thereby inhibiting the accumulation of LDs generated through FAO and enabling TAMs to acquire tumorigenic properties 142. A subset of high-lipid TAMs derived from monocytes exhibit elevated levels of lipid uptake and, through interaction with CAFs, activate the inflammatory CXCL12-CXCR4 axis for monocyte recruitment in TNBC, subsequently reprogramming monocytes to suppress T cell activation 143. Another subset of lipid-associated macrophages displays increased expression of FA transporters and lipid receptors, demonstrating immunosuppression and enhanced phagocytosis, which correlate with poor prognosis in patients with BC 144. LDs within TAMs regulate FA catabolism by inducing mitochondrial respiration; targeted inhibition of mTOR can prevent mitochondrial respiration induced by LDs, thus abolishing the immunosuppressive effect exerted by TAMs 141. The uptake of distinct lipids by the immune cells in the BC microenvironment mediates multiple immunomodulatory effects. Epidemiological studies have revealed that the consumption of polyunsaturated FAs reduces infiltration by macrophages and neutrophils, while increasing infiltration by T cells and CD3+ lymphocytes to modulate the BC microenvironment, exerting anti-tumor activity, and prolonging survival 145.

High levels of FAs and cholesterol also activate NK cells, and the accumulation of cholesterol in these cells can trigger their anti-tumor activity. However, the immunosuppressive microenvironment of BC directly exacerbates the phenotype of NK cells and disrupts their function 146. In mouse BC models, CD36 and peroxisome proliferators mediate lipid accumulation in NK cells. This leads to downregulation of perforin and granzyme expression, resulting in an inhibitory effect on NK cell metabolism and impaired transport processes 147, 148. Additionally, the dysregulation of lipid metabolism leads to lipid accumulation and downregulation of major histocompatibility complex expression in DCs. Lipid accumulation induces endoplasmic reticulum stress in DCs along with the activation of Xbox-binding protein 1 splicing reaction factor, which enhances triglyceride biosynthesis and ultimately impairs antigen presentation, contributing to immune evasion by BC cells 149, 150. The MDSCs in the BC microenvironment exhibit similar functions. G-CSF and GM-CSF derived from tumor cells can induce the expression of lipid transport receptors in tumor-infiltrating MDSCs through STAT3/5 signaling, resulting in increased uptake of FAs, and intracellular accumulation of lipids increases the oxidative metabolism of MDSCs and activates the immunosuppressive mechanism 151. This may be related to the type of exogenous FAs ingested. The accumulation of arachidonic acid in MDSCs leads to increased biosynthesis of prostaglandin E2, which mediates MDSC differentiation through E-prostanoid 1/2/4 receptor and inhibits antigen-specific activation of CD4+T cells and CD8+T cells, thus promoting BC progression. MDSC induced by E-prostanoid 2 receptor has stronger inhibitory activity on T cells 152.

##### Adaptive immune cells (CD4+T cells, CD8+T cells, regulatory T cells (Treg cells))

The two primary T cell subsets within the BC microenvironment, helper T cells (CD4+T cells) and cytotoxic T cells (CD8+T cells), rely on lipid metabolism mechanisms to execute their immune functions. For instance, effector T-cells exhibit elevated levels of FA production, whereas Tregs exhibit increased lipolysis and lipid oxidation 153, 154. Naive T cells have limited energy requirements and depend primarily on mitochondrial function and FAO. Effector T cell energy sources expand to meet the heightened ATP demand. However, an abundance of free lipids in the microenvironment detrimentally affects effector T cell functionality. Furthermore, FFAs can promote the recruitment of immunosuppressive cell types such as Tregs and MDSCs. Increased levels of free cholesterol inhibit signal transduction through T cell receptors and impair CD8+T cell function, particularly due to the lack of key enzymes involved in the catabolism of free lipids within CD8+T cells 155, 156. Additionally, AMPK interferes with effector T-cell differentiation by inhibiting mTOR activity while simultaneously enhancing lipid oxidation 157. Moreover, sphingomyelin 2, an essential enzyme in sphingomyelin catabolic metabolism, downregulated in BC. Overexpression of wild-type sphingomyelin 2 increases anti-PD-1 efficacy in mouse models of BC, which is associated with an enhanced Th1 response 158, 159.

T cell senescence is a potent mechanism employed by tumor cells and Tregs to facilitate immune evasion in BC, leading to impaired T cell function within the TME. Aged T cells exhibit dysregulated lipid metabolism, characterized by lipid accumulation and alterations in metabolic enzymes and metabolites. Previous studies have demonstrated that immunoglobulin-like transcript 4, an immunosuppressive molecule expressed in tumor cells, can activate the MAPK-ERK1/2 signaling pathway to enhance FA synthesis and lipid accumulation in tumor cells, thereby inducing effector T cell senescence and inhibiting specific T cell senescence expression. This ultimately enhanced anti-tumor immunity in BC mice 160. Similarly, IVA phospholipase A2 upexpressed in both tumors and T cells has been shown to induce effector T cell senescence 161. Furthermore, the selective activation of LXR specifically within TNBC, impedes the mitochondrial metabolism of CD8+T cells and disrupts cholesterol localization on the plasma membrane. Conversely, blocking LXR activation enhances the cytotoxicity of CD8+T cells for effective tumor eradication 162.

### Heterogeneity within the microenvironment of breast cancer

#### Hypoxia

The proliferation of BC cells leads to increased oxygen consumption and anoxia response beyond the range of oxygen delivery, primarily driven by hypoxia-inducing factor 1α (HIF-1α). This phenomenon is a significant characteristic of the invasive BC microenvironment, which can stimulate angiogenesis, facilitate tumor cell proliferation, inhibit cell apoptosis, and promote immune evasion. Hypoxia complicates the cellular communication and metabolic processes within the microenvironment. Exosomes derived from BC cell lines (MDA-MB-231, BT-474) exert immunosuppressive effects by negatively regulating T cell activity through TGF-β in anoxic conditions, thereby promoting immune escape 163. Wang et al. demonstrated that under glucose deficiency during hypoxic conditions, carbon glutamine serves as an alternative carbon source to support the growth of BC cells by upregulating acetyl-CoA synthase 2 (ACSS2), consequently enhancing FA synthesis 164. The upregulation of ACSS2 is associated with the aggressive behavior of BC 165. HIF-1α down-regulates acyl-CoA dehydrogenase to inhibit FA β-oxidation and prevent acetyl-CoA entry into the tricarboxylic acid cycle (TCA) cycle 166. Additionally, HIF-1α stimulates FA uptake by upregulating CD36 expression and members of the fatty acid-binding protein family (FABP3, FABP4, and FABP7) 167, 168. Upregulated FABP4 coordinates PUFA uptake to maintain LD formation in BC cells under hypoxic conditions and is essential for BC cell survival and energy supply in hypoxic environments. Meanwhile, hypoxia inhibits the activity of SCD1, reoxygenation can restore its activity and increase the synthesis of MUFA, thus promoting the recurrence of BC 78. Ultimately, these alterations in exogenous FA uptake, along with the enhanced de novo synthesis and weakened FAO induced by hypoxia, collectively contribute to LD accumulation and promote BC growth.

#### Low pH

The acidic characteristics of the TME, previously recognized as induced by the adaptive response to poor oxygenation, are attributed to hypoxia-induced anaerobic glycolysis in tumor cells, resulting in excessive lactic acid production and a subsequent pH decrease within the microenvironment. However, further investigations have revealed that tumor cells exhibit a preference for glycolysis over oxidative phosphorylation (OXPHOS) even under sufficient oxygen conditions (the "Warburg effect"), leading to an inadequate oxygen supply for significant lactic acid generation by TCA 169. Although the underlying mechanisms and drivers remain unclear, it is evident that acidic environments significantly affect tumor progression and evolution. It has been reported that low pH conditions can induce alterations in the propensity of long-chain UFAs, leading to up-regulation of desaturase and elongation enzymes expression in MCF-7 BC cell line, wherein the elevated transcript levels of associated proteins serve as a pivotal factor 170. Low extracellular pH is believed to indirectly activate the SREBP pathway via nuclear translocation of SREBP2, thereby triggering activation of SREBP2-associated target genes and augmenting cholesterol biosynthesis 171. This mechanism may be attributed to overexpression of acid-sensing receptor OGR1 on the BC cell membrane, where OGR1 activation can initiate downstream signal transduction through phospholipase C and p-Akt pathways, ultimately inducing LD accumulation 86.

#### Oxidative stress

The body maintains metabolism, immunity, homeostasis, and other regulatory mechanisms through oxidative stress. Excessive harmful stimulation disrupts the balance of the oxidation-antioxidant system, leading to the constant production of highly active molecules, such as ROS, resulting in pathological damage to cells and tissues. Therefore, the pathological mechanism of oxidative stress involves the oxidative damage caused by free radicals. LPO, which results from the direct combination of ROS and UFAs is involved in regulating signal transduction and protein function during inflammation and tumor progression. MDA and other end products play complex roles in tumors. Additionally, the non-enzymatic oxidative modification of proteins induced by MDA influences immune components and regulates immune responses 172, 173. Interestingly, long-term maintenance of low to moderate levels of ROS promotes BC progression, whereas DNA damage leads to genomic instability, which drives carcinogenic mutations 173. High levels of ROS can trigger apoptosis and inhibit the progression of TNBC 174, possibly due to the cytotoxicity associated with high levels of ROS and abnormal cell death mediated by ROS. Relevant studies have shown a significant increase in MDA levels with disease stage progression among patients with BC, along with a significant decrease in antioxidant oxidase levels; the inflammatory cytokines produced are positively correlated with oxidative stress levels and tumor aggressiveness 175. One possible reason for this may be that estrogen-related mechanisms enhance oxidative stress in BC, leading to continuous platelet activation and the release of bioactive storage factors, ultimately promoting proliferation and invasion 176.

Oxidative stress modulates lipid metabolism in BC by regulating protein expression. Endothelial lipase, a cell surface-associated lipase, is abundantly expressed in BC and is significantly associated with shorter metastasis-free survival in node-negative, untreated patients, potentially due to the upregulation of endothelial lipase expression through AMPK activation under severe oxidative stress. This leads to the accumulation of LDs and enhanced BC cell proliferation 177. Overexpression of myoferlin in TNBC regulates epidermal growth factor receptor activity, which is inhibited by oxidative stress, resulting in decreased OXPHOS and ATP levels. Consequently, this disrupts intercellular vesicle communication and alters SFA/UFA ratio, ultimately leading to increased sensitivity to targeted metabolic drugs 178. In conclusion, oxidative stress induces LPO in BC cells, with a bidirectional impact on tumor progression, which may vary across different stages. However, the precise underlying mechanism requires further investigation.

## Enhanced intervention strategies for lipid metabolism in breast cancer

Given the intricate roles of lipid components in the BC microenvironment, BC cells exhibit plasticity during lipid synthesis, storage, and catabolism. Preclinical studies from Global Trial Identifiers website and WHO international clinical trial registration platform have indicated that targeting these processes is a promising therapeutic approach for the treatment of BC (Table 1). Clinical trials have explored the use of statins as anticancer agents by selectively intervening with cholesterol anabolism in patients with BC. Laboratory models have demonstrated that statins reduce membrane cholesterol levels through cytokine binding and disruption of KRAS-mediated PI3K/TBK/AKT signaling, ultimately inducing apoptosis in BC cells 179. Combining nuclear receptor ROR gamma antagonists with statins has shown a synergistic anti-tumor effect, specifically against TNBC 180. Simvastatin inhibits HMGCR expression, downregulates MVA pathway and GPX4 expression, and promotes ferroptosis in TNBC cells. The combination of simvastatin and metformin suppresses endothelin-1 expression, thereby inhibiting hypoxia-inducing factors and alleviating hypoxia while reducing angiogenesis, which is a potential application for BC treatment 181, 182. Furthermore, TVB-2640, an FASN inhibitor that targets FA synthesis, is currently undergoing Phase II clinical trials for HER2+ metastatic BC.

Lipids have emerged as pivotal nutrients in promoting the progression of BC, and the effect of dietary lipids on the treatment response of BC has garnered significant attention from numerous clinical studies. Epidemiological evidence suggests that adherence to a Mediterranean diet may confer potential benefits against BC by directly or indirectly inhibiting proliferation, inducing cell cycle arrest and apoptosis, and reducing immune evasion and angiogenesis through secondary compounds present in olive oil and n-3/n-6 polyunsaturated FAs 183. The ketogenic diet has been identified as a therapeutic anticancer agent, lowering serum insulin levels by upregulating β-hydroxybutyric acid levels in mouse models of BC, thereby inhibiting tumor progression and lung metastasis; when combined with rapamycin (mTOR inhibitor), it exhibits promising anti-tumor effects 184. Intervention experiments have demonstrated that both ketogenic and low-carbohydrate diets significantly improve physical fitness, muscle/fat ratio, and quality of life among patients recovering from BC and that these diets are deemed safe and beneficial 185. Furthermore, a large prospective cohort study revealed an increased risk of BC in participants who consumed fatty fish weekly 186. Phytosterols found naturally in vegetable oils, nuts, and seeds play an important role in reducing p-AKT expression as well as markers associated with metastasis (alkaline phosphatase and matrix metalloproteinases) and angiogenesis (VEGF and CD67) in BC 187. In conclusion, the future development of diverse strategies for intervention at various stages of BC based on the dynamic regulation of lipid metabolism will pose a new challenge.

## Future directions

Considering the tightly interconnected metabolic preferences and the dominant environment of BC origin tissues and tumor cells, LMR is considered to be a significant attribute of BC. However, the mechanism by which dysregulation of lipid metabolism escalates the risk of BC remains incompletely understood. Investigating the distinctions among related molecular subtypes, tissue structural characteristics, and tumor microenvironment backgrounds can enhance the application of hierarchical management and targeted blocking of metabolic pathways. The plasticity of lipid metabolism deserves attention. Among various aspects, identifying lipid metabolic molecules that play a predominant role during the specific stage of development in different BC subtypes is a crucial issue, anticipated to serve as potential diagnostic and therapeutic targets. The dynamic fluctuations in metabolite levels reflect the activity levels of metabolic enzymes, the combination of DNA, RNA, and protein expression profiles can aid in determining the biological function status of BC cells. Whereas the metabolic vulnerability of non-cancer cells within the tumor microenvironment should not be overlooked, thereby potentially limiting the intervention strategies targeting metabolites or metabolic enzymes.

There exists a diverse array of intricate and dynamic interaction networks between BC cells and non-cancerous constituents in the microenvironment. Currently, the majority of lipid-related investigations concentrate on CAFs, CAAs, as well as the metabolic interplay between immune cells and tumor cells. However, we posit that there are undiscovered pathways pertaining to lipid metabolism in cellular components. Consequently, the origin of lipid metabolites in vivo studies may lack clarity, necessitating further exploration into metabolite identification, molecular mechanisms elucidation, and technological advancements to evaluate metabolic heterogeneity. Precise targeting of specific lipid metabolites or enzymes may disrupt the anti-tumor role of immune cells in the breast cancer microenvironment 188. Indeed, metabolic suppression of immune cells, such as cytotoxic T lymphocytes and natural killer cells, can attenuate the effect of other anti-tumor modalities. The metabolic vulnerability of cells can be found using candidate methods. For example, siRNAs, shRNAs, CRISPR-Cas9 gene editing screening can reveal cell type-specific vulnerabilities depending on the cell source 189, and specific carcinogenic mutations may confer selective metabolic vulnerability on tumor cells. Moreover, solely targeting a singular lipid metabolic pathway or specific target may not result in complete eradication of BC cells, nevertheless it is evident that combining therapies such as targeted inhibitors, cytosuppressants known organic compounds and dietary interventions can effectively complement existing strategies for BC treatment.

## Conclusion

LMR is mandatory for the infinite proliferation and metastasis of BC cells. The current evidence suggests that specific lipid metabolism exists with significant variations in the different BC subtypes, driving the abnormal activity and biological behavior of BC cells. There are pleiotropic roles of LMR in remodeling effects on the BC microenvironment, including abundant secretory factors and crosstalk between multicellular components. Meanwhile, lipid availability is limited by the heterogeneity of the microenvironment. Precise control over lipid metabolism rewiring and understanding of plasticity within the BC microenvironment hold promising implications for developing targeted treatment strategies against this disease. Therefore, interventions targeting the lipid metabolism in BC may facilitate innovative advancements in clinical applications.

## Figures and Tables

**Table 1:** Preclinical studies on targeted intervention of lipid metabolism in breast cancer in recent years.

Official Title	Study Type	Intervention	Phase	Sponsor	ID
Survival Benefits of Statins in Breast Cancer Patients With Abnormal Lipid Metabolism	Interventional	Drug: statins Behavioral: Dietary intervention group (control group)	Phase 3	Peking Union Medical College Hospital	NCT03971019
Phase II Trial to Evaluate the Efficacy of the FASN Inhibitor, TVB-2640, in Combination With Trastuzumab Plus Paclitaxel or Endocrine Therapy in Patients With HER2+ Metastatic Breast Cancer Resistant to Trastuzumab-Based Therapy	Interventional	Drug: Anastrozole Drug: FASN Inhibitor, TVB-2640 Drug: Exemestane	Phase 2	Mayo Clinic	NCT03179904
The Effect of Chemotherapy on Fat Metabolism and Digestion and Function in Breast Cancer Patients	Observational	Not Applicable	Not Applicable	Texas A&M University	NCT01890824
A Phase 2 Single Arm Study to Examine the Effects of Metformin on Cancer Metabolism in Patients With Early Stage Breast Cancer Receiving Neoadjuvant Chemotherapy	Interventional	Drug: Metformin	Phase 2	Oxford University Hospitals NHS Trust	NCT01266486
A Study to Evaluate the Effect of Letrozole and Tamoxifen on Bone and Lipids in Postmenopausal Women With Breast Cancer	Interventional	Drug: Letrozole Drug: Tamoxifen	Phase 3	Novartis Pharmaceuticals	NCT00171704
Targeting Triple Negative BREAst Cancer Metabolism With a Combination of Chemotherapy and a Diet Mimicking FASTing Plus/Minus Metformin in the Preoperative Setting: the BREAKFAST Trial	Interventional	Dietary Supplement: Fasting-mimicking diet Drug: Metformin Drug: Preoperative chemotherapy	Phase 2	Fondazione IRCCS Istituto Nazionale dei Tumori, Milano	NCT04248998
Investigating Atezolizumab in Newly Diagnosed Endocrine Receptor Positive Breast Cancer Patients According to Their AdipOsity (AteBrO)	Interventional	Drug: Letrozole Drug: Atezolizumab	Early Phase 1	Universitaire Ziekenhuizen KU Leuven	NCT04630210
Multicenter, Randomized, Phase II Study of Neoadjuvant Chemotherapy Associated or Not With Zoledronate and Atorvastatin in Triple Negative Breast Cancers - YAPPETIZER Study	Interventional	Drug: Zoledronate Drug: Atorvastatin 80mg Drug: Standard neoadjuvant cht	Phase 2	Mario Negri Institute for Pharmacological Research	NCT03358017
The Effects of Adjuvant Zoladex Plus Tamoxifen on Breast Density in Pre- or Peri-menopausal Women With Early-stage Breast Cancer	Interventional	Drug: tamoxifen Drug: Goserelin	Not Applicable	Zhejiang Cancer Hospital	NCT00827307
Is Lipid Composition Mapping Using Magnetic Resonance Imaging an Effective Early Detection Tool for Breast Cancer in High Risk Populations?	Observational	Procedure: Magnetic resonance imaging (MRI)	Not Applicable	University of Aberdeen	NCT03949946
A Phase II Study Of Topical Ceramide Lipids As Treatment For Cutaneous Breast Cancer	Interventional	Drug: ceramide	Phase 2	Alliance for Clinical Trials in Oncology	NCT00008320
Effects of Adjuvant Endocrine Therapy With Aromatase Inhibitors on the Postoperative Lipid Levels in Postmenopausal Breast Cancer Patients	Observational	Drug: Exemestane Drug: Letrozole Drug: Anastrozole	Not Applicable	Chinese Academy of Medical Sciences	NCT02765373
Pre-Surgical "Window of Opportunity" Trial of the Combination of Metformin and Atorvastatin in Newly Diagnosed Operable Breast Cancer	Interventional	Drug: Metformin Drug: Atorvastatin Procedure: Breast surgery	Early Phase 1	Columbia University	NCT01980823
A Phase II Study of Simvastatin in Women at High Risk for a New Breast Cancer	Interventional	Drug: simvastatin	Phase 2	Sidney Kimmel Comprehensive Cancer Center at Johns Hopkins	NCT00334542
A Phase I Prevention Study of Atorvastatin in Women at Increased Risk for Breast Cancer	Interventional	Drug: atorvastatin calcium Other: laboratory biomarker analysis	Phase 1	National Cancer Institute (NCI)	NCT00637481
Lipidomic Characterization in Non-metastatic Breast Cancer Women Undergoing Surgery: a Pilot Study	Interventional	Procedure: Lipidomic analysis for breast cancer patients	Not Applicable	Fondazione Policlinico Universitario Agostino Gemelli IRCCS	NCT06026631
Atorvastatin in Triple-Negative Breast Cancer (TNBC) Patients Who Did Not Achieve a Pathologic Complete Response (pCR) After Receiving Neoadjuvant Chemotherapy, a Multicenter Pilot Study	Interventional	Drug: Atorvastatin Drug: Capecitabine	Phase 2	M.D. Anderson Cancer Center	NCT03872388
Single Arm Phase 2 Study of Metformin and Simvastatin in Addition to Fulvestrant in Metastatic Estrogen Receptor Positive Breast Cancer	Interventional	Drug: Metformin/Simvastatin/Fulvestrant	Phase 2	National University Hospital, Singapore	NCT03192293
Efficacy of Statin Addition to Neoadjuvant Chemotherapy Protocols for Breast Cancer	Interventional	Drug: Pitavastatin Drug: placebo	Phase 2 Phase 3	Mansoura University	NCT04705909
Study of the Therapeutic Effect of Atorvastatin on the Clinical Outcomes in HER2 Negative Breast Cancer Patients"	Interventional	Drug: Atorvastatin 80mg Other: placebo	Phase 2 Phase 3	Beni-Suef University	NCT05103644
Comparative Clinical Study Evaluating the Anti-tumor Effect of Metformin Versus Atorvastatin as an Adjuvant Therapy With Chemotherapy in Patients With Non-metastatic Breast Cancer	Interventional	Drug: Placebo, metformin and atorvastatin	Phase 4	Tanta University	NCT05507398
Dose Optimization of Rosuvastatin in Early Stage and Metastatic Estrogen Receptor Positive Breast Cancer Patients on Endocrine Therapy	Interventional	Drug: Rosuvastatin	Phase 1	Duke University	NCT02483871
A Phase II Study of Neo-Adjuvant Statin Therapy in Postmenopausal Primary Breast Cancer: A Window-of-Opportunity Study	Interventional	Drug: Atorvastatin	Phase 2	Lund University Hospital	NCT00816244
Combination of CAF and Simvastatin Improves Response to Neoadjuvant Chemotherapy and Increases Tumor-Free Margin in Locally Advanced Breast Cancer: A Randomized, Double-Blind, Placebo-Controlled Trial	Interventional	Drug: Simvastatin 40mg Drug: Placebo oral capsule	Phase 2	Indonesia University	NCT04418089
Phase Ib/II Study of EPA-Based EphA2 Targeted Therapy for Patients With Metastatic Triple-Negative Inflammatory Breast Cancer	Interventional	Drug: Dasatinib Dietary Supplement: Icosapent Ethyl	Phase 1 Phase 2	National Cancer Institute (NCI)	NCT05198843
A Randomized Window of Opportunity Study of Preoperative Letrozole and Simvastatin Versus Letrozole Alone in Stage I-III Hormone Receptor Positive, HER2 Negative Breast Cancer	Interventional	Drug: Letrozole Drug: Simvastatin	Early Phase 1	Emory University	NCT05464810
The Effect of Statins on Markers of Breast Cancer Proliferation and Apoptosis in Women With Early Stage Breast Cancer	Interventional	Drug: Simvastatin Other: Laboratory Biomarker Analysis	Phase 2	Michael Simon	NCT03454529
Vimentin Expression-based Therapeutic Response in Triple Negative Breast Cancer Receiving Combination of Simvastatin and NAC: A Randomized, Double-Blind, Placebo-Controlled Trial	Interventional	Drug: Simvastatin 40mg Drug: Placebo	Phase 2	Indonesia University	NCT05550415
Effects of aromatase inhibitors on blood lipids in postmenopausal patients with hormone receptor-positive breast cancer in northern China and prognosis of treatment with different lipid-lowering drugs: study protocol for a prospective, multi-center, open-label, randomized, parallel-group, controlled, two-phase trial	Interventional	Steroidal AI group:Exemestane;Non-steroidal AI group:Letrozole or anastrozole;	Phase 4	The First Hospital of China Medical University	ChiCTR1900024790
Clinical study on the prevalence of blood lipid in the primary breast cancer patients at initial diagnosis, during chemotherapy andafter systematic therapy andits influence on the treatment andprognosis of breast cancer	Observational	Not Applicable	Not Applicable	Department of Endocrine and Breast Surgery of the First Affiliated Hospital of Chongqing Medical University	ChiCTR-OOB-15007261
Effects of endocrine therapy on postoperative lipid mentabolisam in patients with breast cancer	Observational	Not Applicable	Post-market	Cancer hospital Chinese Academy of Medical Sciences	ChiCTR-OCS-13004203
Omega-3 Fatty Acids, Oxylipins, and Tolerance of Aromatase Inhibitor Therapy	Interventional	Dietary Supplement: Omega-3 fatty acid supplement	Phase 2	University of Michigan Rogel Cancer Center	NCT04268134
Randomised Control Cross-over Trial to Test How Dietary Plant Sterols Modify Tumour Promoting Capabilities of Non-tumour Host Cells in Volunteers With Elevated LDL-C	Interventional	Dietary Supplement: Cholesterol Reducing Strawberry Yogurt Drink (Tesco Ltd) Dietary Supplement: Low Fat Strawberry Yogurt Drinks (Morrisons Ltd)	Not Applicable	University of Leeds	NCT04147767
Effects of Mediterranean Diet Based Intervention in Postmenopausal Women With Breast Cancer Receiving Adjuvant Hormone Therapy: the Randomized Controlled Trial	Interventional	Behavioral: Mediterranean diet	Not Applicable	Gangnam Severance Hospital	NCT04045392
Fish Oil and Evening Primrose Oil in the Treatment of Breast Cancer	Interventional	Dietary Supplement: Fish oil + EPO	Not Applicable	University of Belgrade	NCT03516253
Personalised nutrition intervention for breast cancer survivors based on individual molecular analyses (nutrigenetics, lipidomics and microbiomics)	Interventional	Diet Group (D) participants will be provided with a personalized nutritional strategy (diet + supplementation)	Not Applicable	AZTI - Member of the Basque Research & Technology Alliance	ISRCTN13901439

## Abbreviations

ACAT1: Acetyl-CoA acetyltransferase 1
ACC: Acetyl-CoA carboxylase
ACLY: ATP citrate lyase
ACOX2: Acyl-coa oxidase 2
ACSL4: Acyl-coa synthase 4
ACSS2: Acetyl-CoA synthase 2
AMPK: AMP-activated protein kinase
ATGL: Adipose triglyceride lipase
BC: Breast cancer
BCSC: Breast cancer stem cell
CAA: Cancer-associated adipocyte
CAF: Cancer-associated fibroblast
CEMM: Cholesterol-rich membrane microdomains
Cer: Ceramide
CL: Cardiolipin
CPT1A: Carnitine lipoacyltransferase 1A
DC: Dendritic cell
DHEA: Docosahexaenoic acid
DOX: Doxorubicin
Elovl5: Elongation of very long-chain fatty acids protein 5
EMT: Epithelial-mesenchymal Transition
FABP: Fatty acid binding protein
FAO: Fatty acid oxidation
FASN: Fatty acid synthetase
FFAR4: Free fatty acid receptor 4
FFA: Free fatty acid
GPX4: Glutathione peroxidase 4
GSK-3β: Glycogen synthase kinase-3β
HIF-1α: Hypoxia-inducing factor 1α
HMGCR: Hydroxymethylglutarate monoacyl-CoA reductase
HO: Heme oxygenase
HSL: Hormone-sensitive lipase
IGF-1: Insulin-like growth factor 1
IPP: Isopentenyl pyrophosphate
LD: Lipid droplet
LMR: Lipid metabolic reprogramming
LPO: Lipid peroxidation
LXR: Liver X receptor
MAGL: Monacylglycerol lipase
MDA: Malondialdehyde
MDR: Multidrug Resistance
MDSC: Marrow derived suppressor cell
MET: Mesenchymal-epithelial transition
mTORC1: mTOR complex 1
mTORC2: mTOR complex 2
MUFA: Monounsaturated fatty acid
MVA: Mevalonate
NK: Natural killer
Nrf2: Nuclear factor erythroid 2-associated factor 2
OGP: Osteoblastic growth peptide
OGR1: Ovarian cancer G protein-coupled receptor 1
OXPHOS: Oxidative phosphorylation
PC: Phosphatidylcholine
PG: Prostaglandin
PPAR: Proxisome proliferator activated receptor
PUFA: Polyunsaturated fatty acid
ROS: Reactive oxygen species
SCD1: Stearoyl-CoA desaturase 1
SFA: Saturated fatty acid
SM: Sphingomyelin
SPC: Sphingodylcholine
SQLE: Squalene monooxygenase
SREBP1: Sterol regulatory element binding protein 1
TAM: Tumor-associated macrophage
TAX: Paclitaxel
TCA: Tricarboxylic acid cycle
TME: Tumor microenvironment
TNBC: Triple-negative breast cancer
Treg cell: Regulatory T cell
UFA: Unsaturated fatty acid
VAMP3: v-SNARE protein vesicle-associated membrane protein 3
27-HC: 27-hydroxycholesterol
